# Exploring patterns of female house mouse spatial organisation among outbreaking and stable populations

**DOI:** 10.1002/ece3.10843

**Published:** 2024-03-19

**Authors:** Nikki Van de Weyer, Wendy A. Ruscoe, Peter R. Brown, Steve Henry, Freya Robinson, Lyn A. Hinds, Kevin P. Oh

**Affiliations:** ^1^ Applied BioSciences Macquarie University Sydney New South Wales Australia; ^2^ CSIRO Health and Biosecurity Canberra Australian Capital Territory Australia

**Keywords:** home range, invasive alien species, mouse plague, *Mus musculus*

## Abstract

The size and distribution of home ranges reflect how individuals within a population use, defend, and share space and resources, and may thus be an important predictor of population‐level dynamics. Eruptive species, such as the house mouse in Australian grain‐growing regions, are an ideal species in which to investigate variations in space use and home range overlap between stable and outbreaking populations. In this study, we use spatially explicit capture–recapture models to explore if space use and home range overlap among female mice could serve as indicators of changes in population density leading into summer. Additionally, we assess the sensitivity of space use and home range estimates to reduced recapture rates. Our analysis did not reveal variations in the spring spatial organisation of female mice based on existing capture–mark–recapture data. However, our study highlights the need to balance monitoring efforts within regions, emphasising the importance of exploring studies that can improve spatial recaptures by optimising trapping efforts. This is particularly important in Australian agricultural systems, where varying farm management practices may drive differences in population dynamics.

## Graphical abstract

In this study, we use spatially explicit capture–recapture models to explore if space use and home range overlap among female house mice could serve as indicators for changes in population density in Australian grain‐growing regions. Our analysis did not reveal variations in the spring spatial organisation of female mice based on existing capture–mark–recapture data. Nevertheless, our study highlights the need to balance monitoring efforts within regions, emphasising the importance of exploring studies that can improve spatial recaptures by optimising trapping efforts.

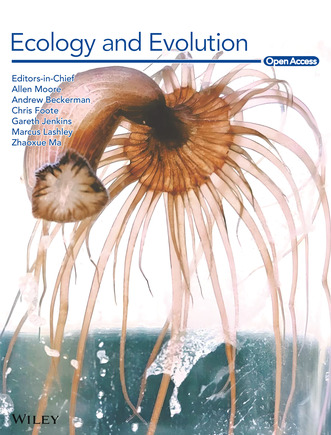

## 1 INTRODUCTION

Home range defines an individual's access to space and resources, and in many species, may both determine and be shaped by interactions with neighbouring conspecifics (Powell & Mitchell, 2012). The size, orientation and distribution of home ranges reflect social and spacing behaviours such as territoriality and co‐operation, which can impact survival and reproduction and have important consequences for population dynamics (Chitty, 1967; Kjellander et al., 2004; Krebs, 2013a). For example, negative density‐dependence in home range size is often interpreted as reflecting greater intraspecific competition with increasing density (Adams, 2001; Efford et al., 2016), highlighting a potential mechanism of population self‐regulation. Examining how space use varies with different population trajectories provides an opportunity to explore hypotheses regarding the impact of social factors on ecological processes (Gaines & Johnson, 1984).

Populations of small mammals that undergo irregular eruptive outbreaks have been the focus of extensive ecological research, yet the degree to which spatial organisation may vary throughout periods of extreme population fluctuation is not well understood (Andreassen et al., 2013). While the triggers of population outbreaks are predominantly attributed to environmental (extrinsic) factors such as food availability, some have hypothesised that a collapse of social structure and density‐dependent spacing could also play a critical role in driving these outbreaks (Eccard et al., 2011; Krebs et al., 1995). Central to this hypothesis is the idea that female territoriality may act as a limiting factor for populations, as dominant females could potentially restrict the reproductive opportunities of individuals without established territories (Lambin & Krebs, 1991; Sutherland et al., 2005). Thus, exploring variations in female space use in spring could be used to provide insights into the social mechanisms that might foreshadow a breakdown or change in density‐dependent processes (Brown et al., 2010; Krebs et al., 1995).

The house mouse (*Mus musculus*) in Australian grain‐growing regions is an ideal model species in which to study the general role of spacing behaviour and population dynamics. Wild house mice undergo irregular fluctuations in numbers, occasionally resulting in ‘plagues’ that cause significant economic damage to all stages of grain crop production (Caughley et al., 1998). While the occurrence of outbreaks has been shown to be closely tied to high winter rainfall, these rain events do not always trigger mouse plagues in all populations within a region, and it is common to observe patchiness in outbreak occurrence at paddock scales (i.e. within a farm). Determining whether spatial organisation is relevant to understanding the heterogeneity of house mouse outbreaks at these scales is becoming increasingly important (Chambers et al., 1996; Ruscoe et al., 2021). One of the first studies to investigate the patterns of space use in Australian wild mouse populations hypothesised that during an outbreak year, favourable extrinsic conditions (i.e. rainfall) enable female mice to establish permanent residency, maintain kin groups and facilitate relatively high reproductive output (Sutherland et al., 2005). Therefore, in the period preceding a population outbreak, the spatial organisation of female mice might be predicted to reflect high levels of co‐operation, characterised by high spatial overlap between neighbouring individuals.

Recent advances in the application of spatially explicit capture‐recapture (SECR) models have enabled researchers to quantify density and space use from live trapping datasets in field studies of small mammals (Bogdziewicz et al., 2016; Johnsen et al., 2019; Romairone et al., 2018). The primary aim of SECR models is to provide accurate estimates of population density by incorporating trap and animal capture locations using capture–mark–recapture (CMR) data, a commonly adopted method of small mammal monitoring (Efford et al., 2016). In addition, SECR models can have a broader application in inferring individual movements (McLaughlin & Bar, 2021), activity patterns (Distiller et al., 2020), space use (Bogdziewicz et al., 2016; Johnsen et al., 2019) and home range overlap (Efford et al., 2016). These studies aimed at understanding spatial organisation of outbreaking small mammal species have successfully used SECR models to provide data on space use, while also being able to describe spatial organisation and population structure.

In this study, we use house mouse capture–mark data to explore whether space use of free‐living house mice in spring could explain variations in population outbreak trajectories in the subsequent summer. Specifically, we investigate whether variations in SECR‐estimated space use and home range overlap among female house mice in spring differed between outbreaking and stable populations. First, we use SECR density estimates to assess changes in population density between spring and summer. We then describe differences in space use and home range overlap parameters for females among our study populations. In addition, we assess the sensitivity of SECR home range overlap parameter estimates to low recapture rates. According to the theory that female kin groups are important for improved reproductive success and survival leading to greater population growth rates (Sutherland et al., 2005), we predict that in the spring preceding an outbreak, female mice with outbreaking trajectories would display a more cooperative spatial pattern characterised by comparatively higher spatial overlap, reflecting low territoriality.

## 2 METHODS

### 2.1 Study sites

**FIGURE 1:**
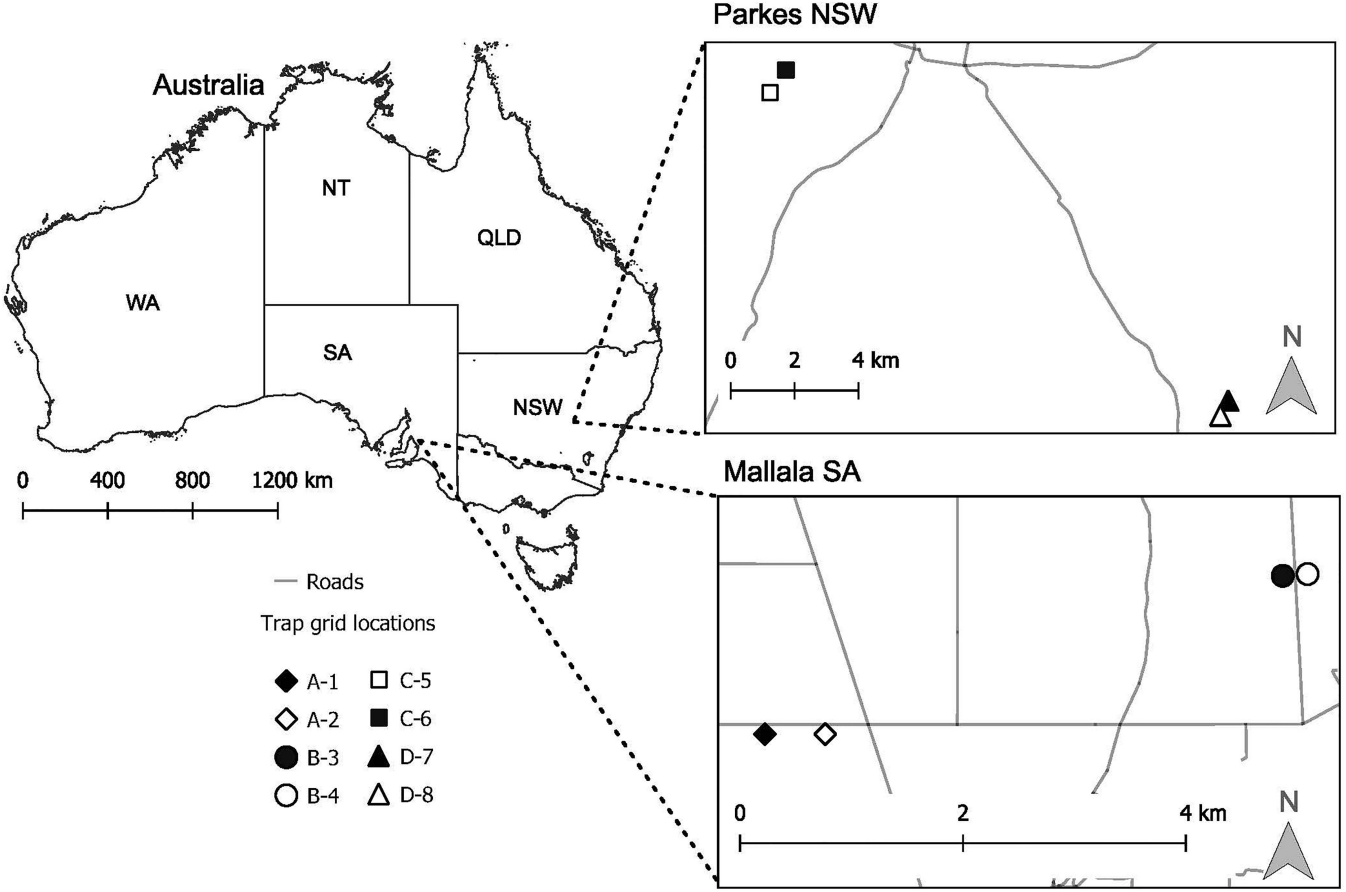
Map of study sites in the Australian grain‐growing regions, showing paddocks A and B near Mallala SA, and paddocks C and D near Parkes NSW. Within each paddock, two independent trapping grids were established and separated by a minimum of 100 m, and maximum 970 m. Map created in QGIS version 3.16.12.

The data used for this study were collected from grain paddocks located near Mallala on the Adelaide Plains, South Australia (SA) (−34°26′59.99″ S, 138°29′59.99″ E) (Figure 1; paddock A and B) and near Parkes, Central West, New South Wales (NSW) (33°8′12.56″ S, 148°10′22.93″ E) (Figure 1; paddock C and D). These paddocks were all farmed using conservation agriculture (zero‐ or no‐till) practices, meaning that paddocks have low soil turnover, with standing stubble remaining in each paddock throughout the study duration. Near Mallala SA, paddocks A and B were wheat paddocks separated by approximately 5 km (Figure 1). This region is predominately used for grain production. Trapping data were collected between October 2019 and February 2020, across four trapping sessions. Near Parkes NSW, the two grain cropping farms (paddock C, barley; paddock D, wheat) were separated by approximately 18 km (Figure 1). This region is also predominantly used for agricultural production. Trapping data used here are from a subset of data collected between November 2020 and February 2021, across four trapping sessions. These data were collected as part of a larger study (Ruscoe, Brown, Henry, et al., 2023), and data have been reanalysed to answer different questions.

### 2.2 House mouse live capture

In each paddock (*n* = 4), two independent live‐capture trapping grids, each separated by a minimum of 100 m, were established (Figure 1) (*n* = 8 trapping grids). On each live‐trapping grid, 64 single capture Longworth traps (25 × 6.5 × 8.5 cm, Longworth Scientific, Abingdon, UK) were set at 10‐m intervals on an 8 × 8 grid. Traps contained polyester fibre bedding and wheat grains for food. They were checked and closed each morning starting at approximately 0630 hours (h) and opened in the evening at about 1700 h. Traps are designed for single capture; however, during this study some traps had multiple captures on a single trap night.

For each study region, Mallala SA during 2019/2020 and Parkes NSW during 2020/2021, live trapping sessions were conducted simultaneously on each trap grid at intervals of 2–6 weeks between sessions. Each trapping session consisted of 4–5 consecutive trap nights, referred to as occasions. To estimate population density, trapping sessions were categorised as spring or summer based on the Australian seasons that are grouped by calendar months that considers the lag in heating and cooling patterns. In Mallala SA, we assigned three sessions (October, November and early December 2019) to spring and one (February 2020) to summer. In Parkes NSW, there were two spring sessions (October and early December 2020), and two summer sessions (January and February 2021) for each trap grid. We chose to classify the early December trapping sessions in each region as spring due to their occurrence in early December aligning with similar transitional spring weather patterns.

Each trapped mouse had a radio frequency identification passive integrated transponder (PIT) tag (MiniHPT8 8 × 1.4 mm; Biomark, Inc.) inserted subcutaneously between the scapulae allowing individuals to be uniquely identified through time. For each trapped individual, we recorded data on trap location, PIT tag identification number, weight, length and sex. The animal was then released at the point of capture. All data were collected under the approval of the CSIRO Wildlife, Livestock, and Laboratory Animal Ethics Committee under the approved application AEC 2019–2018.

### 2.3 Spatially explicit capture–recapture models

Models were fitted using maximum likelihood spatially explicit capture–recapture (ML SECR) models, using the ‘secr’ package (Efford, 2023) in R v4.2.2 (R Core Team, 2022). One limitation to using likelihood estimation‐based SECR models for this study is that they have not yet been extended for use with single‐capture traps. Single‐capture traps do not meet the assumption of capture independence; however, Efford et al. (2009) conducted simulations and found no bias in density estimates for single‐capture traps if less than 86% of traps were occupied. Trap occupancy in each session and on each trap grid in our study never exceeded 86% (see Table 1). We therefore fitted the models using the likelihood for multi‐capture traps. To define the habitat mask (a component of SECR models, see Efford, 2023), a buffer width of 80 m was used with points spaced 5 m apart within the habitat mask.

**TABLE 1:** Summary of house mouse trapping data, including the maximum percentage of traps occupied recorded during any session on each trap grid during spring and summer seasons.

Trap grid	Max. trap occupancy (%)	Spring						Summer					
		Sessions	Detections	Number of individuals				Sessions	Detections	Number of individuals			
				M	F	Unk	Total			M	F	Unk	Total
Mallala SA													
A‐1	39	3	133	57	53	2	112	1	63	19	16	1	36
A‐2	32	3	139	62	47	1	110	1	51	19	18	0	37
B‐3	72	3	59	22	22	1	45	1	232	64	109	1	174
B‐4	69	3	85	39	24	3	66	1	145	49	61	5	115
Parkes NSW													
C‐5	63	2	117	57	30	0	87	2	265	94	94	3	191
C‐6	63	2	140	71	39	1	111	2	367	147	156	5	308
D‐7	36	2	72	44	15	0	59	2	192	66	86	1	153
D‐8	38	2	60	38	10	1	49	2	220	64	109	2	175

*Note*: Data are presented as the total number of sessions, total number of detections (captures), and the number of individuals trapped and marked (M = male, F = female, Unk = unknown sex) for each trap grid.

All models were fitted using the hazard half‐normal detection function. We maximised the likelihood with respect to *D* (population density), λ_0_ and *k*_*p*_. The detection parameter λ_0_ describes the decline in the cumulative hazard of detection with increasing distance from a detector (i.e. here a single Longworth trap). We chose parametrised models using *k*_*p*_, rather than σ (a linear measure of spatial scale), where *k*_*p*_ is defined by $k_{p}=\sigma_{p}\surd D_{p}$. The parameter *k*_*p*_ allows for density‐dependant σ, which provides insights into the degree of home range overlap, while *p* discerns between populations (see Efford et al., 2016).

We considered six multi‐session SECR models, each model included session‐specific density, where sessions were defined by the trap grid and the corresponding session (i.e. A1.1, A1.2). We allowed the detection parameter λ_0_ to vary by either region or sex. The region effect considered that λ_0_ varied between sampling years (Mallala SA 2019/2020, and Parkes NSW 2020/2021), while a sex effect assessed if one sex was more detectable than the other, by using the ‘group’ argument in the ‘secr’ package. Finally, we investigated whether a constant, sex or both sex and population trajectory provided a more explanatory model for the parameter *k*_*p*_. Population trajectory, defined by the paddock's summer population trajectory (outbreaking or stable) and the corresponding season, allowed us to account for paddock, and season‐specific variation in density‐dependent σ. Models were compared using Akaike information criterion (AIC) corrected for small sample size (∆AIC) (Burnham & Anderson, 2002). Estimates from the best‐fit model were back‐transformed to a biological scale using the predict function in the ‘secr’ package.

To confirm if populations were increasing (outbreaking) or stable, we estimated the percent change in density between spring and the following summer, where $\text{percent change}=\frac{D_{\text{summer}}-D_{\text{spring}}}{D_{\text{spring}}}\times 100$. Populations with a high percent increase in density (>100%) between spring and summer were considered outbreaking.

### 2.4 Home range overlap parameters *k* and S_95_

By allowing the parameter *k*_*p*_ to vary, we were able explore sex and paddock level differences in space use. We used estimates from the best‐fit model to calculate the corresponding density‐dependent σ for females in each paddock by $\sigma_{p}=k_{p}\surd D_{p}$. To describe female home range overlap in spring, we used the parameter *k*_p_ for females to calculate S_95_ defined by $S_{95}=6\pi k^{2}$, which represents an estimate of the number of individual females within the area of another female's 95% home range limits (Efford et al., 2016).

### 2.5 Sensitivity of home range parameters to recapture rates

To assess the sensitivity of density‐dependent space use (σ) and home range overlap (S_95_) to recapture rates, we conducted repeated random sampling within each trapping session and trap grid at reduced recapture rates of 20% (*n* = 5 times each). Where a 20% reduction in the recapture rate is equivalent to one night of trapping effort. We then reran the best‐fit model (as detailed above) using the modified trapping datasets. Using estimates for females from each model, we calculated the relative bias of the corresponding σ estimate to describe the difference from the original dataset.

## 3 RESULTS

### 3.1 Live captures

The total number of captured individuals declined in paddock A (trap grids A‐1 and A‐2) between spring and summer, and increased in paddocks B, C and D (Table 1). Recapture rates were low across all trap grids and sessions. During spring, detections per individual ranged from 0.7 to 0.9 and in summer ranged from 0.6 to 0.8 across all trap grids.

### 3.2 House mouse density

The best‐fit model, with 25 parameters, included sex‐specific variations in detection probability (λ_0_) and allowed the parameter *k*_p_ to vary for each sex and population outbreak trajectory (D ~ season, λ_0_ ~ sex, k ~ sex + trajectory; see Table 2). Spring density estimates derived from the best‐fit model were notably lower (Table 3) than the total number of individuals trapped (Table 1).

**TABLE 2:** Spatially explicit capture–recapture (SECR) model selection table based on Akaike Information Criterion (AIC).

Model			npar	logLik	∆AIC	AIC weight
D	λ_0_	*k*				
Season	Sex	Sex + trajectory	25	−8312.33	0	1
Season	Region	Sex + trajectory	24	−8344.74	62.77	0
Season	Sex	Sex	22	−8514.76	398.699	0
Season	Region	Sex	21	−8529.06	425.248	0
Season	Sex	1	20	−8534.96	435.006	0
Season	Region	1	19	−8867.72	1098.484	0

*Note*: The ‘npar’ column denotes the number of fitted model parameters.

**TABLE 3:** SECR parameter estimates from the best‐fit model (D ~ season λ_0_ ~ sex k ~ sex + trajectory).

Parameter		Trajectory	Spring	Summer	Percent change
			Estimate	Estimate	
λ_0_	Male	n.a	0.07 (0.01)		
λ_0_	Female	n.a	0.13 (0.01)		
λ_0_	Unknown	n.a	0.01 (0.00)		
k	Female	Stable	33.5 (1.7)	32.6 (2.3)	
k	Male	Stable	47.6 (2.3)	46.3 (3.2)	
k	Female	Outbreaking	29.7 (1.3)	52.7 (2.0)	
k	Male	Outbreaking	42.1 (1.8)	74.8 (2.8)	
D	A1	Stable	35.1 (3.7)	36.6 (7.0)	4.39
D	A2	Stable	27.4 (2.8)	50.3 (12.0)	83.38
D	B3	Outbreaking	11.2 (1.8)	227.7 (24.3)	1940.58
D	B4	Outbreaking	29.7 (4.5)	162.2 (26.7)	446.66
D	C5	Outbreaking	55.8 (8.7)	230.6 (30.5)	313.39
D	C6	Outbreaking	61.0 (8.0)	305.9 (29.5)	401.18
D	D7	Outbreaking	29.6 (4.5)	155.8 (20.4)	427.27
D	D8	Outbreaking	47.7 (10.9)	134.2 (13.7)	181.30

*Note*: D refers to density per hectare, the parameter k relates to the degree of home range overlap and λ0 is the baseline detection rate. Trajectory is defined by the paddock's summer population density (outbreaking or stable). Percent change is the difference between spring and summer population density for each trap grid. Values represent the mean (SE).

### 3.3 Spring space use and home range overlap

**FIGURE 2:**
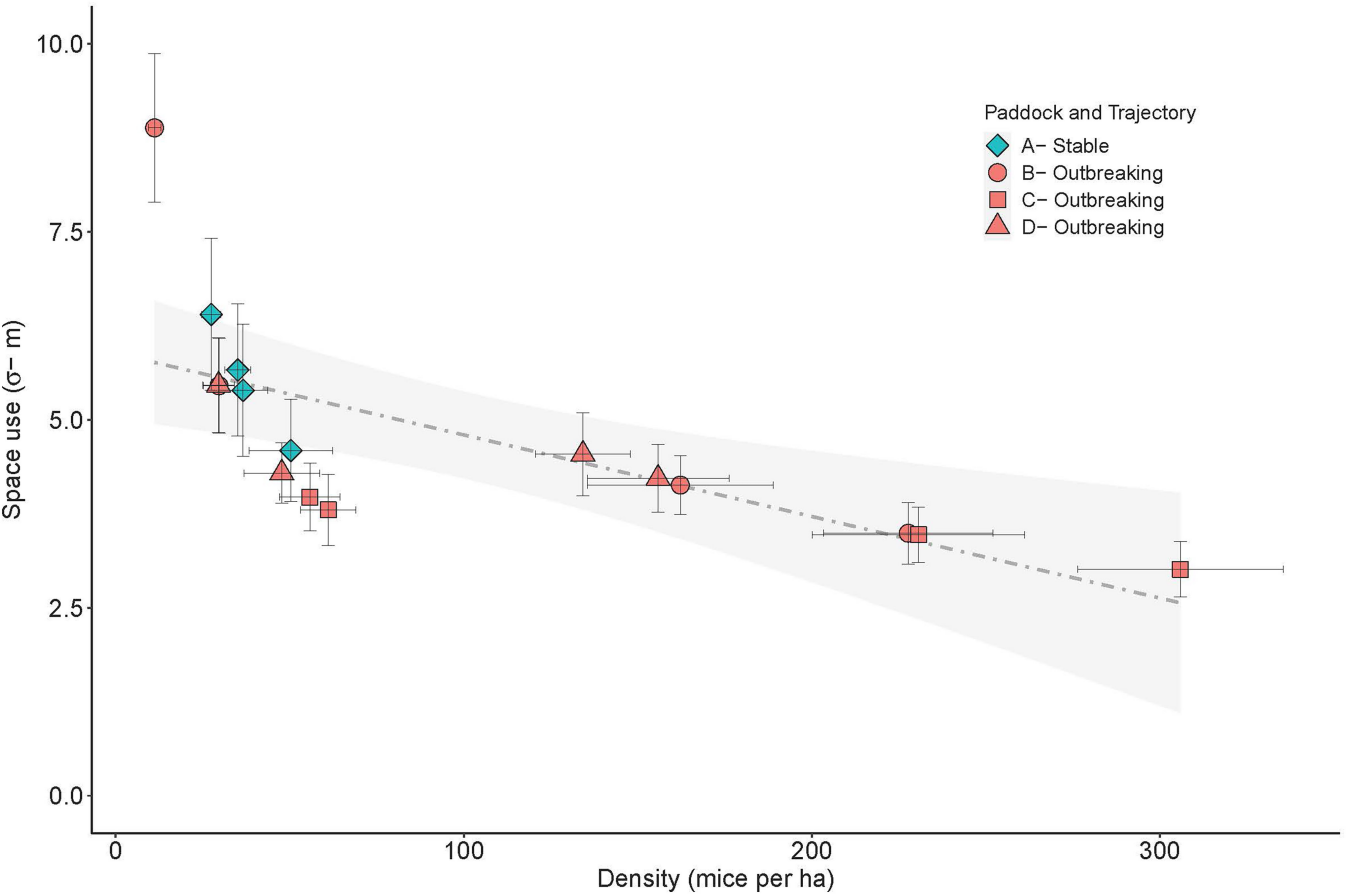
Relationship between density‐dependent σ for females and mouse density (mice per ha) based on estimates from the best‐fit model (*D* ~ season, λ_0_ ~ sex, k ~ sex + trajectory) for each paddock. Shapes represent session‐specific estimates, with colours indicating stable (blue) and outbreaking (orange) summer population trajectories. Error bars are ±1 SE. The trend line illustrates the linear relationship between density‐dependent σ ~ density, while grey shading is the 95% predictive interval.

Incorporating outbreak trajectory (outbreaking or stable) into the model, showed an improvement in model fit (∆AIC = 0; Table 2). Calculated density‐dependent σ for females decreased with increasing density (Figure 2). During spring, σ for females was comparatively similar between paddocks at similar densities. The corresponding S_95_ for females in outbreaking populations in spring was 1.6 and in populations with stable summer population trajectories was 2.1. These values indicate an overlap of two other females within another female's 95% home range limit. In summer, female S_95_ estimates increased to 5.2 and remained consistent in the stable population (S_95_ = 2.0) in summer.

### 3.4 Sensitivity of space use parameters to low recapture rates

**FIGURE 3:**
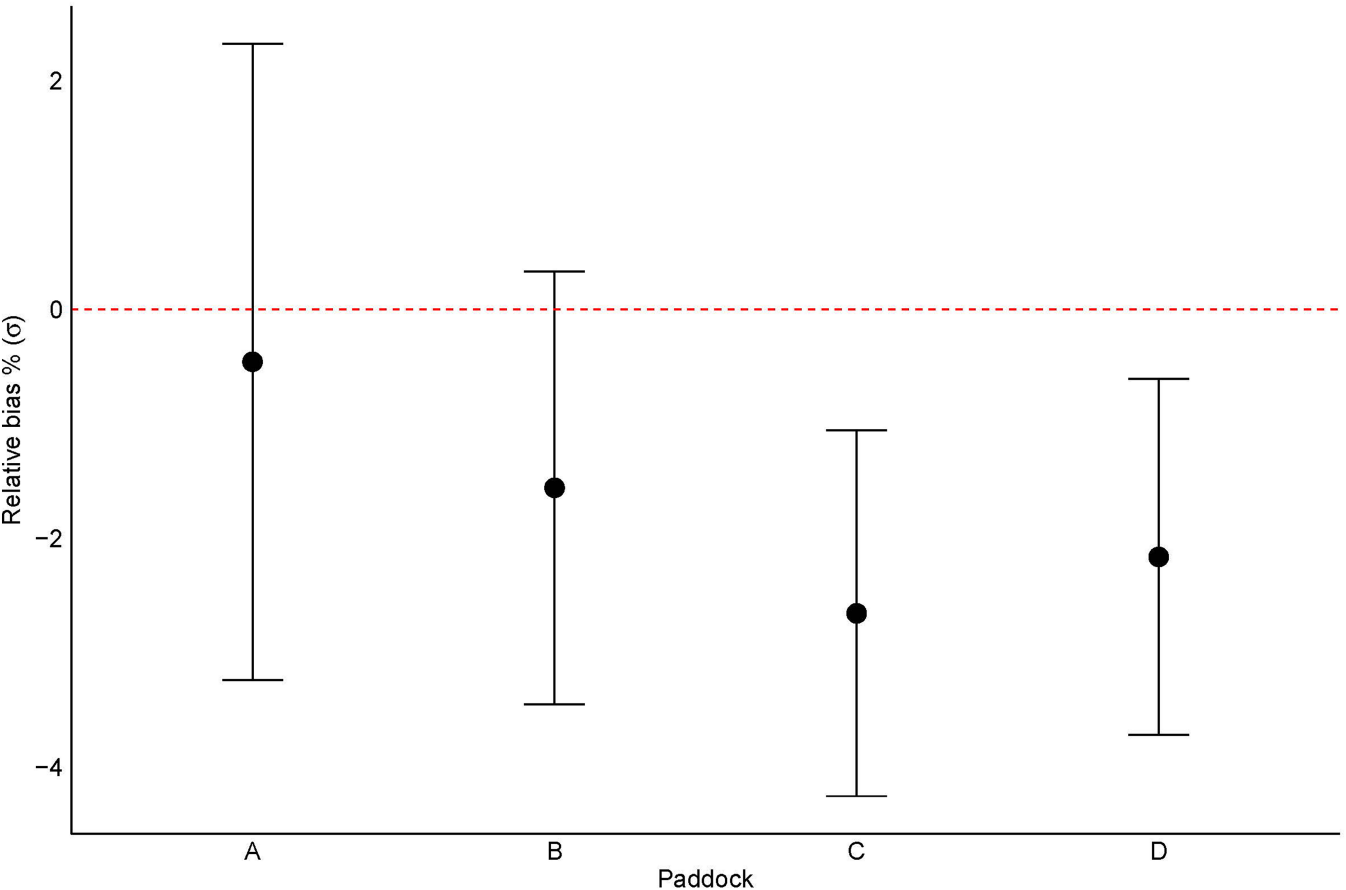
Relative bias (%) of density‐dependent σ for females in each paddock. Recaptures were randomly reduced by 20% (*n* = 5 times). Points represent the mean relative bias across each reduced model rerun and error bars are ±1 SE. The red dotted line at 0 represents the deviation from the original dataset.

Reducing recaptures rates by 20% (equivalent to one night reduction in trapping) indicated that, on average, density‐dependent σ estimates for females were lower than our original values (Figure 3). In paddock C and D, relative bias was overall lower than the original dataset. Calculated spring S_95_ values for females were similar to the original dataset values (mean S_95_ values for stable = 2.7 and outbreaking = 2.07). The reduction in recapture rates did not introduce any conversion issues when using the modified datasets to fit the best‐fit model (D ~ season, λ_0_ ~ sex, k ~ sex + trajectory).

## 4 DISCUSSION

In this study, we explored whether differences in space use and home range overlap among female mice during spring could serve as indicators of changes in population density leading into summer. Building upon earlier predictions by Sutherland et al. (2005), we predicted that female mice in outbreaking populations would display a more cooperative spatial pattern characterised by comparatively higher home range overlap in spring. Despite the improvement in model fit with the inclusion of population trajectory, spring estimates of density‐dependent space use (σ) and home range overlap (S_95_) for females were comparatively similar for outbreaking and stable populations.

Here, we used density‐dependant σ and S_95_ to explore spring space use between female mice in populations with distinct summer population trajectories. These parameters can, however, have limitations when inferring home range overlap, as they assume a circular range of activity (Efford et al., 2016), that may not perfectly represent the irregularly shaped home ranges typically observed in free‐living house mice (Chambers et al., 2000). Simulation studies examining the sensitivity of spatial estimates highlighted potential challenges associated with elongated or irregular home ranges, which may produce negative errors (Ivan et al., 2013). However, SECR density estimates appear to be robust even when dealing with non‐circular home ranges, especially when detectors (i.e. traps) are spread in two‐dimensions (Efford, 2019), as in the trap grid array used in our study. While the robustness of SECR density estimates is supported by existing literature, less is known about the sensitivity of the parameter *k*_p_ and σ to the assumption of circular home ranges, especially in species like house mice who are likely to have spatial dependencies (i.e. burrow or nest sharing) (Gerber & Parmenter, 2015). Studies should, therefore, consider variations in detection probabilities and incorporate individual variation at the trap level when modelling σ (Gerber & Parmenter, 2015). This emphasises the importance of viewing space use and home range overlap estimates not as absolute values but as a tool that might be able to provide insights into important ecological questions.

Another limitation to our study is that the capture–mark–recapture data were collected as part of a larger project, meaning trap locations, sessions and occasions may not have been optimal for collecting spatial recapture data intended for use with SECR models. Our sensitivity assessment (Figure 3) revealed potential implications for estimates of *k*_*p*_. Our analysis suggested a negative bias in the corresponding σ estimate for females in each paddock when compared to our original dataset. Therefore, owing to our low spatial recapture rates, initial estimates of σ could be prone to underestimation. Species like the house mouse, known for inherently have low recapture rates (Krebs et al., 1994), are particularly susceptible to the underestimation of space use parameters unless recapture rates can be improved. Optimising trap locations, grid layout and trapping effort as observed in another study on common voles (*Microtus arvalis*) (Romairone et al., 2018) could significantly improve spatial recaptures for mice. Therefore, future studies incorporating SECR models should explore trapping methods aimed at improving spatial recapture data for wild house mice in similar systems.

While we were unable to detect differences in spring space use, home range overlap and hence, territoriality between our study populations, other mechanism(s) that might contribute to changes or a breakdown in density‐dependant space use still require further investigation. One potential explanation for the differences in summer population trajectories in our study is the degree of genetic similarity between neighbouring females in spring. The formation and benefits of kin groups have been observed in barn populations of house mice (Evans et al., 2020; König et al., 2012), yet there are still limited data on the genetic structure of free‐living house mice at a paddock scale, despite evidence suggesting that kin groups may be critical in facilitating breeding leading into an outbreak (Sutherland et al., 2005). If kin groups are important for triggering an outbreak through reproductive success, then females in our study populations with outbreaking trajectories would have higher relatedness between neighbouring individuals (Hamilton, 1964; König et al., 2012). While we did not directly measure relatedness in this study, relatedness between neighbouring individuals should be explored in future studies to provide a more comprehensive understanding of the role of kinship in changes in population trajectories.

From a management perspective, our study offers valuable insights into the importance of balancing monitoring location and trapping effort (Freeman et al., 2022; Gerber & Parmenter, 2015) within grain cropping regions. In addition, the low SECR spring density estimates observed in our study and knowledge on the tendency of SECR models to, on average, underestimate density highlight the importance of refining approaches to mouse population monitoring and density estimation (Gerber & Parmenter, 2015). These considerations are important as inaccurate density estimates may lead to suboptimal decision‐making in resource allocation, potentially reducing the effectiveness of outbreak intervention strategies.

In conclusion, although we were unable to detect differences in spring spatial organisation of female mice using existing trapping data, the use of capture–mark–recapture data still provides an accessible means of collecting spatial information on a broader scale and across a larger number of individuals when compared to alternative methods. Our analysis highlights the importance of balancing monitoring efforts within regions, particularly for house mice in agricultural systems where varying farm management practices may drive differences in population dynamics. It is essential to acknowledge the limitations of SECR estimates in our study, which are sensitive to the number of recaptures. Future studies exploring adequate trapping efforts and design are necessary to increase spatial recaptures and minimise estimate biases.

## AUTHOR CONTRIBUTIONS

**Nikki Van de Weyer:** Conceptualization (equal); data curation (lead); formal analysis (equal); investigation (equal); methodology (equal); project administration (equal); resources (equal); software (equal); validation (equal); visualization (equal); writing – original draft (lead); writing – review and editing (equal). **Wendy A. Ruscoe:** Conceptualization (equal); data curation (equal); investigation (equal); methodology (equal); project administration (equal); writing – review and editing (equal). **Peter R. Brown:** Conceptualization (equal); data curation (equal); funding acquisition (lead); investigation (equal); methodology (equal); project administration (equal); supervision (supporting); writing – review and editing (equal). **Steve Henry:** Conceptualization (equal); data curation (equal); methodology (equal); project administration (equal); resources (equal); writing – review and editing (equal). **Freya Robinson:** Data curation (equal); methodology (equal); project administration (equal); resources (equal); validation (equal); writing – review and editing (equal). **Lyn A. Hinds:** Conceptualization (equal); investigation (equal); writing – review and editing (equal). **Kevin P. Oh:** Conceptualization (equal); investigation (equal); methodology (equal); project administration (equal); supervision (lead); validation (equal); visualization (equal); writing – original draft (supporting); writing – review and editing (equal).

## FUNDING INFORMATION

The analysis and research were supported by an Australian Government Research Training Program (RTP) Scholarship (20212897). Field data used in this project were collected under a larger project funded by the Grains Research and Development Corporation (CSP1806‐015RTX) and supported by CSIRO Health & Biosecurity.

## CONFLICT OF INTEREST STATEMENT

The authors declare there are no conflicts of interest.

## Data Availability

The raw trapping data used in this study are openly available in Dryad under the DOI: 10.5061/dryad.3tx95x6n8.
